# Reduced expression of PMCA1 is associated with increased blood pressure with age which is preceded by remodelling of resistance arteries

**DOI:** 10.1111/acel.12637

**Published:** 2017-08-09

**Authors:** Robert Little, Min Zi, Sally K. Hammad, Loan Nguyen, Alexandra Njegic, Sathishkumar Kurusamy, Sukhpal Prehar, Angel L. Armesilla, Ludwig Neyses, Clare Austin, Elizabeth J. Cartwright

**Affiliations:** ^1^ Division of Cardiovascular Sciences Manchester Academic Health Science Centre The University of Manchester AV Hill Building Manchester M13 9PT UK; ^2^ School of Food Science and Nutrition The University of Leeds Leeds LS2 9JT UK; ^3^ Department of Biochemistry Faculty of Pharmacy Zagazig University Zagazig 44519 Egypt; ^4^ Research Institute in Healthcare Science School of Pharmacy University of Wolverhampton Wolverhampton WV1 1LY UK; ^5^ University of Luxembourg Avenue de l'Universite Esch‐sur‐Alzette L‐4365 Luxembourg; ^6^ Faculty of Health and Social Care Edge Hill University Lancashire L39 4QP UK

**Keywords:** arterial remodelling, ATP2B1, blood pressure, hypertension, plasma membrane calcium ATPase

## Abstract

Hypertension is a well‐established risk factor for adverse cardiovascular events, and older age is a risk factor for the development of hypertension. Genomewide association studies have linked *ATP2B1*, the gene for the plasma membrane calcium ATPase 1 (PMCA1), to blood pressure (BP) and hypertension. Here, we present the effects of reduction in the expression of PMCA1 on BP and small artery structure and function when combined with advancing age. Heterozygous PMCA1 null mice (PMCA1^Ht^) were generated and conscious BP was measured at 6 to 18 months of age. Passive and active properties of isolated small mesenteric arteries were examined by pressure myography. PMCA1^Ht^ mice exhibited normal BP at 6 and 9 months of age but developed significantly elevated BP when compared to age‐matched wild‐type controls at ≥12 months of age. Decreased lumen diameter, increased wall thickness and increased wall:lumen ratio were observed in small mesenteric arteries from animals 9 months of age and older, indicative of eutrophic remodelling. Increases in mesenteric artery intrinsic tone and global intracellular calcium were evident in animals at both 6 and 18 months of age. Thus, decreased expression of PMCA1 is associated with increased BP when combined with advancing age. Changes in arterial structure precede the elevation of BP. Pathways involving PMCA1 may be a novel target for BP regulation in the elderly.

**Abbreviations**
BP: blood pressure
CSA: cross‐sectional area (of arterial wall)
NA: noradrenaline
NCX: sodium–calcium exchanger
PMCA: plasma membrane calcium ATPase
VSM: vascular smooth muscle
VSMC: vascular smooth muscle cell
W:L: arterial wall thickness to lumen diameter ratio

## Introduction

Cardiovascular diseases are the world's leading cause of morbidity and mortality. Hypertension is a major modifiable risk factor for adverse cardiovascular events including stroke and aneurysm, and for heart and renal failure (Kearney *et al*., 2005). It is thought that at least one in five people worldwide have elevated blood pressure (BP) and that high BP contributes to around 9 million deaths worldwide annually (Mancia *et al*., 2013; Mozaffarian *et al*., 2016).

Around 90% of people with hypertension suffer from essential, also known as primary, hypertension (Mancia *et al*., 2013), for which there is no single or clearly identifiable cause. The prevalence of essential hypertension increases with age, in a roughly linear relationship (Buford, 2016), with around 60% of people over 70 years of age being hypertensive (Buford, 2016). With an aging global population, it is projected that, by 2025, around 1.56 billion people will be hypertensive (Kearney *et al*., 2005). Thus, it is now ever more important to understand the underlying basis of BP control and the factors which may increase the risk of developing hypertension with aging.

Genomewide association studies (GWAS) have shown *ATP2B1,* the gene for plasma membrane calcium ATPase 1 (PMCA1), to be highly associated with BP and with essential hypertension, and importantly, demonstrated this as a consistent observation in different ethnic populations (Cho *et al*., 2009; Levy *et al*., 2009; Tabara *et al*., 2010). PMCA1 is a member of the P‐type family of membrane ATPases, which actively extrude Ca^2+^ ions from cells. Of the four members of this family (four separate genes) PMCA1 and PMCA4 are expressed in virtually all tissues and cell types in the body (Strehler *et al*., 2007; Cartwright *et al*., 2011), including the vasculature (Szewczyk *et al*., 2007; Kobayashi *et al*., 2012). A direct association between PMCA1 and BP has been demonstrated in animal studies where a reduction in the expression of PMCA1 has been shown to elevate BP (Kobayashi *et al*., 2012; Shin *et al*., 2013). However, the effect of PMCA1 on BP with advancing age is unknown.

Vascular resistance is determined primarily by small precapillary arteries, less than 300 μm internal diameter (in humans) (Mulvany & Aalkjaer, 1990; Heagerty *et al*., 2010). It is well established that increased total peripheral resistance is the principal contributor to maintaining elevated BP (Heagerty *et al*., 2010). Enhanced vascular contractility is evident in mice with reduced arterial expression of PMCA1 (Shin *et al*., 2013). Whilst this indicates that PMCA1 may play some role in increased vascular resistance, there is a wealth of evidence to support the importance of structural changes of resistance arteries in hypertension (Rizzoni *et al*., 2003; Heagerty *et al*., 2010). Rearrangement of vascular smooth muscle cells (VSMCs) around a smaller lumen diameter without any global change in the arterial wall cross‐sectional area, termed eutrophic inward remodelling, has been shown to be a conventional small artery structural abnormality in patients with chronic essential hypertension (Mulvany & Aalkjaer, 1990; Rizzoni *et al*., 2003; Heagerty *et al*., 2010; Little *et al*., 2016). Such changes in small artery structure have strong prognostic significance in hypertensive patients, over and above all other known cardiovascular risk factors (Rizzoni *et al*., 2003; Mathiassen *et al*., 2007b). Antihypertensive therapies which reduce both BP and reverse remodelling of resistance arteries have been shown to significantly reduce cardiovascular risk compared to interventions which reduce BP without affecting arterial structure (Buus *et al*., 2013). It remains an important topic of debate whether the remodelling is a consequence of hypertension or whether it precedes its development (Bakker *et al*., 2002; Martinez‐Lemus *et al*., 2004; Izzard *et al*., 2006). Hence, it is important to understand the mechanisms which underpin eutrophic inward remodelling, and the relationship between elevated BP and this remodelling.

Therefore, we sought to determine how PMCA1 may be involved in arterial structure and function with aging. We herein present that global heterozygous deletion of PMCA1 in mice is associated with an age‐dependent elevation of BP with development of inward eutrophic remodelling and that structural changes in mesenteric resistance arteries occur before a detectable increase in BP.

## Results

PMCA1^Ht^ and PMCA1^flox/flox^ controls (WT) were born in the predicted ratio given that PMCA1 total knockouts are embryonic lethal (Okunade *et al*., 2004), with neither male nor female PMCA1^Ht^ mice displaying reduced fertility compared to WT. PMCA1^Ht^ mice were indistinguishable by eye from WT, appeared healthy and appropriately active, and had similar lifespan to WT animals.

**Figure 1:**
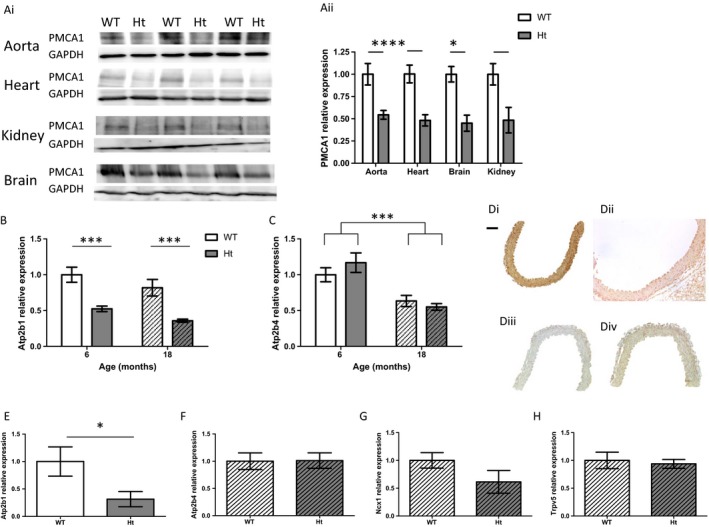
PMCA1^Ht^ mice display reduced PMCA1 expression. (A) Protein expression of PMCA1 was reduced in tissues from 6‐month‐old PMCA1^Ht^ mice. Ai. Western blots (PMCA1 at 143KDa and GAPDH at 50KDa) and Aii. quantification of PMCA1 normalized to GAPDH in WT and Ht mice. *n *= 3 & 3. (*t*‐test for each tissue). (B) Aortic mRNA expression of *Atp2b1*, presented relative to young WT, was significantly reduced in 6‐month‐old (*n *= 8 & 7) and in 18‐month‐old (*n *= 4 & 6) PMCA1^Ht^ mice (*P* < 0.0001). No significant effect of age or interaction was determined (*P* = 0.050 and *P* = 0.925 respectively, 2‐way ANOVA). (C) Aortic mRNA expression of *Atp2b4*, presented relative to young WT, was not significantly reduced in 6 months (*n *= 8 & 7) or in 18‐month‐old (*n *= 4 & 6) PMCA1^Ht^ mice (*P* = 0.714), although a significant effect of age was determined (*P* = 0.0003, 2‐way ANOVA). (D) Detection of primary antibody against PMCA1 in aorta from 6‐month‐old WT Di. and PMCA1^Ht^ Dii. mice. Diii. Staining of aorta when primary antibody omitted. Div. Staining of aorta with DAB reagent only. Scale bar represents 100 μm. E. mRNA expression of *Atp2b1* was significantly reduced in endothelial cells isolated from lungs of 6‐month‐old PMCA1^Ht^ mice compared to cells from age‐matched WT mice (*n *= 3 & 5). (F‐H) Total kidney mRNA expression of *Atp2b4* (E.), *NCX1* (F.) and *TRPV5* (G.) was not significantly different in 18‐month‐old PMCA1^Ht^ mice vs. age‐matched WT animals. All data were plotted as mean ± SEM. *P < 0.05, **P < 0.01, ***P < 0.001.

Immunoblot analysis of tissues from 6‐month‐old animals showed that PMCA1 protein expression was reduced by 45–55% in the aorta, heart, brain and kidney of PMCA1^Ht^ mice compared to WT controls (Fig. 1A). The mRNA level of *Atp2b1* was significantly reduced in the aorta of 6‐month‐old PMCA1^Ht^ mice and also in aorta of 18‐month‐old PMCA1^Ht^ mice compared to aged matched WT animals (Fig. 1B). Reduced expression of PMCA1 had no significant effect on the mRNA level of *Atp2b4* (gene for PMCA4 protein) in aorta from 6 months (*P *= 0.672) or 18‐month‐old (*P *= 0.503) animals, although the relative *Atp2b4* mRNA level was found to be significantly reduced with age (Fig. 1C). PMCA1 was detected throughout the aorta by immunohistochemical staining, with staining being of a lower intensity in the aorta of PMCA1^Ht^ mice compared to those from age‐matched WT animals (Fig. 1D). The mRNA level of *Atp2b1* was significantly reduced in lung endothelial cells of 6‐month‐old PMCA1^Ht^ mice compared to age‐matched WT animals (Fig. 1E). Reduced expression of PMCA1 had no significant effect on the mRNA level of other genes involved in Ca^2+^ homoeostasis including *Atp2b4* (*P *= 0.959), NCX1 (*P *= 0.161) and TRPV5 (*P *= 0.731) in kidney from 18‐month‐old animals (Fig. 1F,G,H, respectively, *t*‐test with Welch's correction).

**Figure 2:**
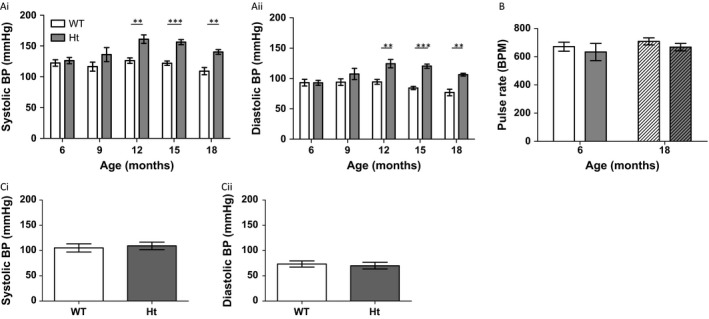
Conscious BP increases with age in PMCA1^Ht^ mice. (A) Conscious peripheral BP (Systolic, Ai. and diastolic, Aii.) of PMCA1^Ht^ mice is significantly higher at 12 months and then at all older ages (*t*‐test vs. age‐matched WT,* n *= 5–7). (B) No significant difference in conscious pulse rate is recorded in young or aged PMCA1^Ht^ mice (*P* = 0.290, and *P* = 0.972 for interaction. Two‐way ANOVA) *n *= 5, 5, 7 & 6. (C) Arterial BP (Systolic, Ci. and diastolic, Cii.) of 6‐month‐old animals, under anaesthesia, does not significantly differ between WT and PMCA1^Ht^ mice (*t*‐test, *n *= 10 & 12). Mean value ± SEM. **P < 0.01, ***P < 0.001.

Conscious peripheral systolic BP and diastolic BP of 6‐ and 9‐month‐old PMCA1^Ht^ mice were not significantly different to those of age‐matched WT mice; however, PMCA1^Ht^ mice aged 12 months and older had significantly elevated peripheral BP when compared to age‐matched WT animals (Fig. 2A). No significant differences in pulse rate were found between 6‐month‐old and 18‐month‐old PMCA1^Ht^ and WT animals (Fig. 2B). Central BP of 6‐month‐old animals, measured under anaesthesia, did not significantly differ between WT and PMCA1^Ht^ mice (Fig. 2C). At 18 months of age, no significant changes in cardiac function of PMCA1^Ht^ mice, and no significant difference in heart size or cardiomyocyte cell area was found compared to age match WT mice (Fig. S1).

**Figure 3:**
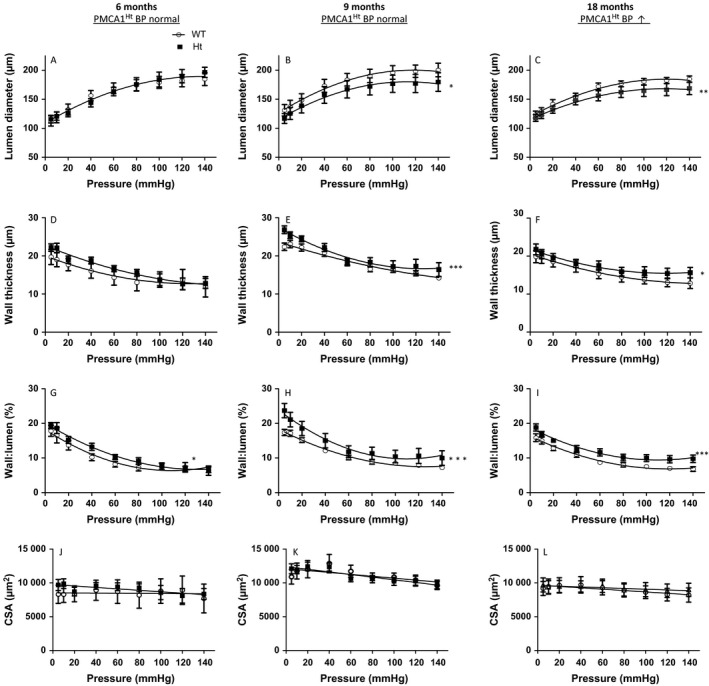
Remodelling of small mesenteric arteries occurs with age in PMCA1^Ht^ mice and before a detectable increase in BP. (A) The internal lumen diameter does not significantly differ between passive mesenteric arteries from WT and PMCA1^Ht^ mice at 6 months of age but is significantly smaller in PMCA1^Ht^ mice at 9 and 18 months (B & C). (D) Wall thickness is similar at 6 months of age and significantly increases with age in PMCA1^Ht^ arteries (E & F). (G, H. and I) The vessel wall thickness to lumen ratio (W:L) is significantly increased in arteries from PMCA1^Ht^ mice aged 6, 9 and 18 months old. (J, K and L) The cross‐sectional area (CSA) of the vessel wall is not significantly different between arteries taken from WT and PMCA1^Ht^ mice at any age tested. Extra sum of squares F‐test analysis was performed. All data were plotted as mean value ± SEM. *n *= 4–8 *P < 0.05, **P < 0.01, ***P < 0.001.

As structural remodelling of resistance arteries is of key prognostic importance in essential hypertension (Rizzoni *et al*., 2003), we assessed the passive properties of isolated mesenteric arteries. Arteries from 6‐month‐old PMCA1^Ht^ mice showed no significant difference in lumen diameter, wall thickness or cross‐sectional area (CSA) (Fig. 3A,D,J, respectively) compared to age‐matched WT mice which also exhibited a similar level of BP. However, the calculated wall to lumen ratio (W:L) was significantly greater in PMCA1^Ht^ mice compared to WT at this age (Fig. 3G). Importantly, arteries from 9‐month‐old PMCA1^Ht^ mice, also ‘normotensive’, displayed significantly reduced lumen diameter and significantly increased wall thickness and W:L (Fig. 3B,E,H, respectively) compared to arteries from age‐matched WT mice. CSA was not significantly different between genotypes at this age (one line adequately fits both datasets) (Fig. 3K). Further, arteries from PMCA1^Ht^ animals with elevated BP (18 months) exhibited a significantly reduced lumen diameter (Fig. 3C) and significantly increased wall thickness and W:L (Fig. 3F,I, respectively) with no significant change in CSA (one line adequately fits both datasets Fig. 3L).

**Figure 4:**
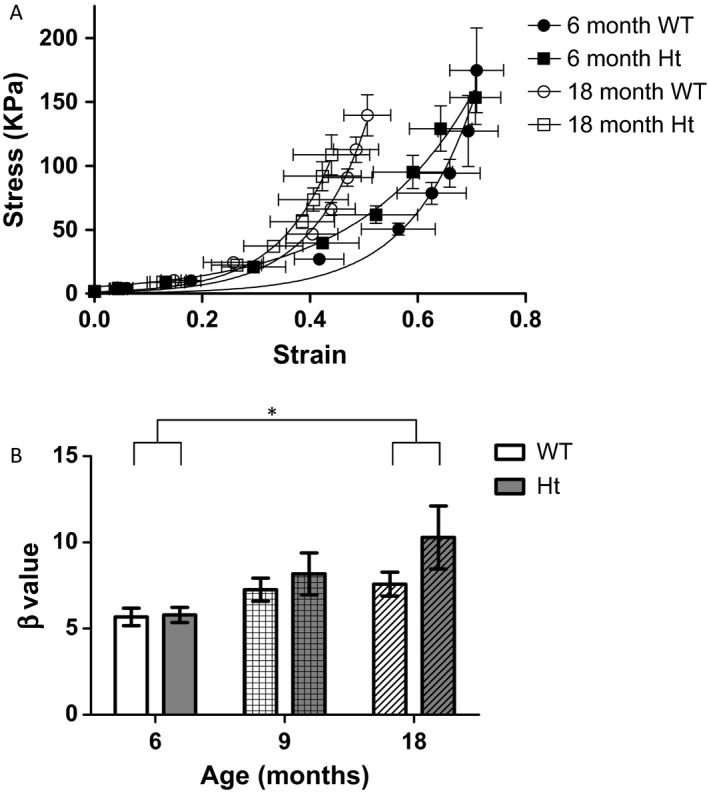
Stiffness of resistance arteries increases with age but is not significantly altered in arteries from PMCA1^Ht^ mice. (A) Stress–strain relationship derived from passive properties shows leftward shift for arteries from 6‐month‐old mice to 18‐month‐olds. (B) The distensibility of arteries from PMCA1^Ht^ mice is not significantly different (*P* = 0.215) from arteries of WT mice as shown by incremental elastic modulus. A significant increase in age was detected (*P* = 0.022) between arteries from 6‐ and 18‐month‐old animals. No significant interaction was determined (0.326) when analysing these 2 ages. Two‐way ANOVA with Bonferroni post hoc test. Mean ± SEM. *n *= 5–8. *P < 0.05.

The distensibility of mesenteric arteries decreased with age, evidenced by a leftward shift in the stress–strain relationship from 18‐month‐old mice relative to arteries from 6‐month‐old mice, but was not modified by reduction in PMCA1 expression (Fig. 4A). The incremental elastic modulus (β) of arteries from PMCA1^Ht^ and age‐matched WT mice was not significantly different at each age (Fig. 4B); however, compared to arteries from 6‐month‐old mice, arteries from 18‐month‐old mice displayed a significantly increased β value, indicative of reduced arterial distensibility (Fig. 4B).

**Figure 5:**
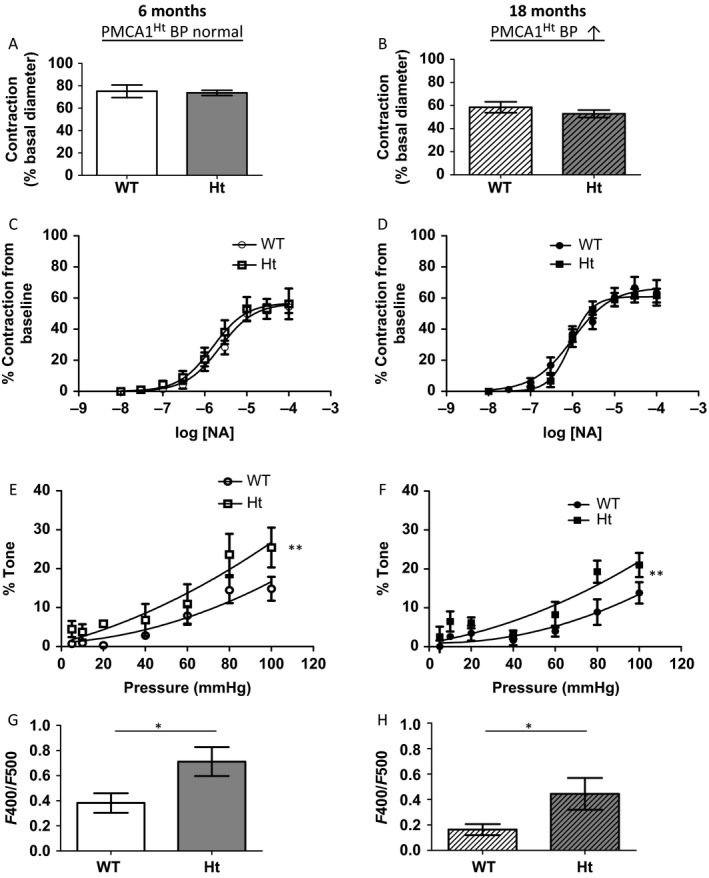
Arteries from PMCA1^Ht^ mice contract similarly but display elevated myogenic tone and basal intracellular calcium. (A & B) The magnitude of contraction to a depolarizing stimulus (100 mM K^+^) is not significantly different for arteries from PMCA1^Ht^ mice for both young (A. *P* = 0.773. *n *= 6 & 7) and aged (B. *P* = 0.334. *n *= 7 & 6) animals (*t*‐test). C & D. Arteries from PMCA1^Ht^ mice do not display a significant difference in contractility to noradrenaline (NA) from both young (C. *P* = 0.537. *n *= 6 & 6) and aged (D. *P* = 0.162. *n *= 4 & 6) animals (Nonlinear regression comparison of fits). (E and F) Arteries from PMCA1^Ht^ mice display significantly elevated myogenic tone in both young (E. *n *= 5 & 5) and aged (F. *n *= 6 & 6) animals (comparison of fit analysis). G. & H. A significantly elevated arterial indo‐1 F_400_/F_500_ emission ratio, indicative of intracellular free Ca^2+^ concentration, was detected at both ages (G & H, young and old, respectively. *n *= 5 & 7 and 7 & 5). All data were plotted as mean value ± SEM. *P < 0.05, **P < 0.01.

Whilst arterial structure clearly has an important influence on BP regulation, we also sought to determine whether PMCA1 may mediate functional changes in resistance arteries. The magnitude of arterial contraction in response to 100 mm K^+^ was not significantly different between PMCA1^Ht^ and WT mice in arteries from either 6‐month‐old (Fig. 5A) or 18‐month‐old (Fig. 5B) mice. The cumulative dose–response profile to NA was very similar for arteries from both 6‐month‐old (LogEC_50_ WT: −5.606 ± 0.140, Ht: −5.806 ± 0.138, Fig. 5C) and 18‐month‐old (LogEC_50_ WT: −6.017 ± 0.242, Ht: −6.038 ± 0.115, Fig. 5D) PMCA1^Ht^ and WT mice. Subsequent to contraction induced with a single maximal dose of NA (30 mm), simultaneous superfusion with the vasodilator acetylcholine (ACh) induced no significant difference in the maximal magnitude of relaxation for vessels from aged PMCA1^Ht^ and WT mice (32.03 ± 10.45% and 39.68 ± 14.54% respectively, *P *= 0.699. *n *= 4 & 5, *t*‐test). Arteries from both WT and PMCA1^Ht^ mice were found to develop tone in Ca^2+^ containing conditions as seen by a significant divergence in the pressure–diameter relationship compared to the nominally Ca^2+^ free condition (Fig. S2). Calculated basal tone was found to be significantly increased in arteries from 6‐month‐old (Fig. 5E) and 18‐month‐old (Fig. 5F) PMCA1^Ht^ mice. The basal F_400_/F_500_ emission ratio, indicative of arterial [Ca^2+^]i (Austin & Wray, 1995), was found to be significantly increased in arteries from PMCA1^Ht^ mice compared age‐matched WT animals at both 6 (Fig. 5G) and 18 months (Fig. 5H) of age.

## Discussion

This study shows that reduced expression of PMCA1 in mice correlates with elevated BP and small artery remodelling when combined with aging. Importantly, we show that arterial remodelling precedes the development of elevated BP. Thus, we propose that reduced expression of PMCA1 predisposes to the development of elevated BP when combined with aging, suggesting that a PMCA1‐mediated mechanism could be targeted to reduce the burden of high BP in older age.

PMCA1 has been described as having a highly important housekeeping function as evidenced by embryonic lethality of global homozygous knockout mice (Okunade *et al*., 2004). GWAS studies showing *ATP2B1* to be highly associated with BP (Cho *et al*., 2009; Levy *et al*., 2009; Tabara *et al*., 2010) have highlighted the potential clinical relevance of *ATP2B1*. The importance of PMCA1 in BP regulation has been demonstrated in previous studies using tissue‐specific ablation and silencing of *Atp2b1* (Kobayashi *et al*., 2012; Shin *et al*., 2013). Our study shows that although reductions in global PMCA1 expression are not associated with significant alteration in BP of 6‐month‐old mice, with advancing age increased BP is revealed.

Aging has been shown to be a key contributor to cardiovascular disease risk (Buford, 2016). Less than 10% of people under 30 years of age have essential hypertension; however, it is estimated that around 60% of people aged over 70 have elevated BP (Buford, 2016). It is clear that both natural aging and genetic factors increase the risk for the development of hypertension and that risk factors may combine to enhance the prospect of adverse cardiovascular events or outcome (Buford, 2016). The results of this study suggest that *Atp2b1* may be an important risk factor for the development and progression of hypertension. As even relatively small (5 mmHg) increases in BP are associated with increased risk of adverse cardiovascular events and mortality (Lewington *et al*., 2002; Tabara *et al*., 2012) managing risk factors for hypertension can have a large positive effect on a population's health.

This study utilized conscious BP measurement to assess the role of PMCA1. This method has the advantage of enabling repeated measures to be undertaken so a longitudinal study can be conducted. It has previously been shown that volume–pressure recording (Coda 6 system) of systolic BP closely agrees with measurements recorded simultaneously by radio telemetry (Feng *et al*., 2008). Elevated BP has previously been reported in mice with reduced PMCA1 expression at 3 months of age, but BP at other ages was not investigated (Fujiwara *et al*., 2014). The basis of our study was to directly generate mice globally heterozygous for PMCA1, and we have utilized Cre‐recombinase under the CMV promoter. Fujiwara and colleagues have produced mice globally heterozygous for PMCA1 using a Cre‐loxP and flippase recombination enzyme–flippase recognition target recombination system under the Tie2 promoter (Fujiwara *et al*., 2014); hence, reduced expression of PMCA1 was engineered differently to our PMCA1^Ht^ mice. Whilst they do not quantify protein levels, Fujiwara and colleagues report approximately 70% reduction in *Atp2b1* mRNA in brain, heart and aorta (Fujiwara *et al*., 2014), whereas our PMCA1^Ht^ mice display approximately a 50% reduction in gene and protein expression of PMCA1 from the aorta compared to WT animals. Therefore, a potentially greater degree of reduced PMCA1 expression may explain Fujiwara and colleagues observed BP phenotype in 3‐month‐old mice designated heterozygous for *Atp2b1*; akin to complete ablation in VSMCs (Kobayashi *et al*., 2012). In contrast, we report no significant difference in peripheral, and also central, BP between WT and PMCA1^Ht^ mice at 6 months of age. Therefore, despite both models being termed global heterozygotes, any direct comparison should be made with caution.

Complete genetic ablation of *Atp2b1* specifically in VSMCs has been reported to be associated with reduced *Ncx1* expression and with a significant increase in *Atp2b4* expression (Kobayashi *et al*., 2012). However, in the present study we report no significant change in the expression of *Atp2b4* in aorta of PMCA1^Ht^ mice or in gene expression of renal Ca^2+^ pumps and channels *Atp2b4*,* Ncx1* or *Trpv5* which are involved in transcellular reabsorption of Ca^2+^. The expression of other PMCA family members, isoforms 2 and 3, has been shown to be limited to specific tissues (Strehler *et al*., 2007), and we have no knowledge of these gene products being detected in the vascular system. Therefore, we suggest the effects we observed on BP and arterial remodelling in our PMCA1^Ht^ mice are unlikely to be strongly due to the expression of NCX1 or related PMCA family members.

Inward eutrophic remodelling of resistance arteries, the rearrangement of existing wall material around a smaller lumen (Heagerty *et al*., 2010), is a key feature of essential hypertension and has been shown to be prognostic for elevated BP and associated with increased adverse cardiovascular risk (Rizzoni *et al*., 2003; Mathiassen *et al*., 2007b). Here, we show significantly reduced lumen diameter, significantly increased wall thickness and no significant change in arterial wall cross‐sectional area from aged PMCA1^Ht^ mice, indicative of inward eutrophic remodelling. Importantly, inward eutrophic remodelling has been described as a feature of accelerated aging (Harvey *et al*., 2015). Strategies to at least slow the progression of vascular remodelling have clinical benefit in reducing BP and reducing the risk of adverse cardiovascular events in older people (Mathiassen *et al*., 2007a; Mulvany, 2012; Harvey *et al*., 2015).

Traditionally arterial structural changes were thought to be adaptive responses to elevated BP (Folkow, 1982); however, others have demonstrated, in genetically modified animals, that vascular remodelling may precede an increase in BP (Zacchigna *et al*., 2006). Whilst there are clearly numerous factors which may influence the relationship between BP and the vasculature, there is continuing debate concerning remodelling as a cause or effect of BP changes (Izzard *et al*., 2006). Our observations from PMCA1^Ht^ mice show evidence for inward eutrophic remodelling in mesenteric resistance arteries before a detectable increase in BP. At 9 months of age, significant changes in lumen diameter, wall thickness and W:L ratio were evident but with no significant increase in conscious peripheral BP. Although we observed no significant changes in lumen diameter or wall thickness in arteries from 6‐month‐old PMCA1^Ht^ mice, we did observe a significant increase in the W:L ratio, which does point to vascular changes occurring. This suggests that the remodelling, at least in part, is a relatively early feature of essential hypertension and is not a direct consequence of a prolonged and significant rise in BP in PMCA1^Ht^ mice. Reductions in arterial distensibility have been reported during normal physiological aging (Mitchell *et al*., 2004) and were demonstrated in the present study. However, this effect was not augmented by reduction in PMCA1 expression. This observation correlates with the proposal that eutrophic remodelling can occur independently of vascular stiffening (Intengan & Schiffrin, 2001). Therefore, we propose that elevated BP in aged PMCA1^Ht^ mice is not directly due to increased vascular stiffness but rather a direct PMCA1‐associated mechanism of eutrophic remodelling.

There is now a body of evidence to show that chronic vasoconstriction can induce eutrophic remodelling (Bakker *et al*., 2002; Eftekhari *et al*., 2007). In the present study, we show that, although contractile responses to high K^+^ solution or to NA are similar in arteries from control and PMCA1^Ht^ mice at both 6 and 18 months of age, basal intrinsic tone is significantly increased in arteries from PMCA1^Ht^ mice. Furthermore, this enhanced tone is evident at 6 months of age suggesting it precedes the development of high BP. This supports previous reports showing that the basal tone of rat mesenteric arteries is enhanced during the development of hypertension (Izzard *et al*., 1996). However, there remains debate about how vascular tone may contribute to BP regulation during the established phase of hypertension. Spontaneously hypertensive rats (SHR) with chronically elevated BP (20 weeks old) have been shown to display no significant increase in mesenteric arterial tone compared to age‐matched ‘normotensive’ animals (Izzard *et al*., 1996), whereas basal tone was increased in middle cerebral arteries from 24‐week‐old (established hypertension) but not 4‐week‐old (prehypertensive) SHR (Gonzalez *et al*., 2008). These contrasting observations may reflect vascular bed differences or the calibre of artery studied. Previous studies have shown that silencing *Atp2b1* increases myogenic tone (Shin *et al*., 2013) and here, we show that reductions in the expression of PMCA1 are associated with enhanced basal tone. The observation of elevated tone in arteries from young and aged PMCA1^Ht^ mice, compared to age‐matched WT animals, is consistent with the notion that PMCA1 may contribute to the development and maintenance of hypertension. Herein, we show a small reduction in the magnitude of basal tone with age, an effect which has been previously noted in mesenteric arteries and in arterioles isolated from the gastrocnemius and soleus muscles of rats (Kang *et al*., 2009; Vessieres *et al*., 2016). Such an age‐related change has not been associated with an elevation of BP in rats (Kang *et al*., 2009), comparable to aging WT mice used in this study. Of note we did not detect a significant effect of age on *Atp2b1* mRNA levels in the aorta. As such, we propose that reduced PMCA1 expression not age *per se* contributes to relative enhanced arterial tone.

Previous studies have shown that PMCA1 can regulate total [Ca^2+^]_i_ in vascular smooth muscle (Kobayashi *et al*., 2012). In support of this, we show here that resistance arteries from PMCA1^Ht^ mice pressurized to 60 mmHg exhibit a significant elevation of the indo‐1 F_400_/F_500_ emission ratio, indicative of [Ca^2+^]_i_. This is consistent with the notion that in PMCA1^Ht^ mice, PMCA1 is modulating basal tone via effects on Ca^2+^ homoeostasis. At present, the molecular pathways underlying remodelling of resistance arteries remain unclear. Further assessment of how Ca^2+^‐dependent pathways may contribute to the development and maintenance of high BP would be useful in designing future targeted treatment strategies for hypertension.

In summary, we show that PMCA1^Ht^ mice develop increased BP with advancing age. In this animal model, age‐dependent increases in BP are preceded by inward eutrophic remodelling of resistance arteries, and elevations in arterial basal [Ca^2+^]_i_ and intrinsic tone. Although we cannot completely exclude the fact that changes in the expression of PMCA1 in other resistance arteries or nonvascular tissues may influence BP, an area for further investigation, we propose that effects of PMCA1 on the resistance vasculature play an important role in the development of hypertension with aging. The results of our study show that changes in the expression of PMCA1, which in younger mice does not significantly influence BP, does so when combined with aging, which is a well‐established risk factor for hypertension and cardiovascular disease (Buford, 2016). The combination of all loci highly significantly associated with BP in GWAS has been reported to account for only up to 10% of the total estimated genetic component for BP (Tabara *et al*., 2012). Therefore, we propose that some of the ‘missing heritability’ for BP can be derived from an interaction between a genetic factor and aging. Changes in the expression and/or activity of PMCA1 may predispose to the development of hypertension. Therefore, PMCA1‐mediated mechanisms can be a target for potentially regulating abnormal BP with age, particularly relevant as the percentage of older people in the population is increasing.

## Experimental procedures

### PMCA1^Ht^ mice

Loss of PMCA1 was engineered using a Cre‐LoxP system, targeting sites flanking exon 2 in *Atp2b1* containing the ATG transcription site. The targeting vector and mice were commercially generated by GenOway (Lyon, France). The targeting vector with two loxP sites flanking exon 2 of *Atp2b1* was transfected into 129Sv/Pas ES cells. Fully validated ES cells clones were injected into C57Bl/6J blastocysts, and the resulting male chimaeras were bred to generate homozygous PMCA1 flox mice (PMCA1^f/f^). To generate a constitutive deletion of PMCA1, PMCA1^f/f^ mice were mated with mice expressing Cre under the CMV promoter (CMV‐Cre C57Bl/6J). Mice were maintained on a mixed genotype background and bred as brother/sister matings.

All animals were maintained in a pathogen‐free facility, housed under 12 h‐light/dark cycle with *ad libitum* access to food and water. Studies were performed in accordance with the UK Home Office and institutional guidelines. All experiments in this study utilized male mice between 6 and 18 months of age. DNA was extracted from a sample of ear tissue for genotyping of each animal as described in Data S1.

### BP measurement

Mice were regularly handled in our experimental facility following weaning. Conscious BP was measured by determining the tail blood volume with a volume–pressure sensor and an occlusion tail‐cuff (Coda System, Kent Scientific) following a 3‐day acclimatization period for the animals. Restrained animals were placed on a warming platform set to 37°C. Blood flow to the distal tail was occluded with a maximal cuffing pressure of 250 mmHg and then steadily deflated over 15 s for a single cycle. Systolic and diastolic pressures were automatically recorded during cuff deflation as blood flowed into the tail. Twenty continuous cycles were performed, with accepted values (volume ≥15 μL from calm and relaxed animals) from the latter ten cycles used for data analysis. 5 seconds between each cycle was programmed. Central BP was recorded, following intraperitoneal injection of Avertin (250 mg/kg body weight), by insertion of a high‐fidelity pressure–volume catheter (Millar Instruments, Houston, Texas, USA) into the right carotid artery. All BP experiments were performed between 09:00 and 12:00 h.

### Echocardiography

Under isoflurane anaesthesia transthoracic two‐dimensional and M‐mode echocardiography were performed using an Acuson Sequoia C256 system (Siemens) as previously described (Mohamed *et al*., 2016).

### Dissection of tissues

Mice were killed by cervical dislocation. The entire mesenteric bed and thoracic aorta were removed and placed separately into ice‐cold HEPES buffer of composition (in mM) 127 NaCl, 5.9 KCl, 1.2 MgSO_4_, 10 HEPES, 11.8 glucose, 2.4 CaCl_2_ at pH 7.4. Fat and adherent tissue was removed from the aorta and the tissue immersed in 4% paraformaldehyde solution for 60mins or immediately frozen in liquid nitrogen. Excised hearts were drained of blood, weighed and either flash frozen or immersed in 4% paraformaldehyde for 24 h. Brain and kidney tissue were also flash frozen. All frozen tissues were stored at −80°C until required. Lung tissue was excised into DMEM growth media (Sigma‐Aldrich) supplemented with 10% (v/v) foetal calf serum (Gibco) and 1% (v/v) penicillin–streptomycin.

### Mouse lung endothelial cell (MLEC) isolation

MLECs were isolated from 6‐month‐old WT and 6‐month‐old PMCA1^Ht^ mice as previously described (Oblander *et al*., 2005; Baggott *et al*., 2014).

### Western blot analysis

Proteins were extracted, separated and transferred as previously described (Mohamed *et al*., 2016). Sufficient protein extract was yielded by extracting from half of the heart, half of one kidney and one hemisphere of the brain from individual animals, whilst aortic proteins were extracted from aortae pooled from six to ten animals. Membranes were probed with monoclonal antibodies against PMCA1 or GAPDH (Abcam) at 1 μg mL^−1^ or 0.2 μg mL^−1^ respectively, overnight at 4°C or after 3 h at room temperature. Hydrogen peroxide‐conjugated secondary antibodies were detected using ECL detection reagents (Amersham).

### Quantitative PCR (qPCR)

Tissues were homogenized by hand under TRIzol Reagent (Invitrogen) with nucleic acids extracted by phenol–chloroform precipitation and eluted in diethylpyrocarbonate (DEPC)‐treated deionized water. Total RNA was extracted from MLEC as previously described (Baggott *et al*., 2014). Total RNA was treated with amplification grade DNAse 1 (Sigma‐Aldrich) and quantified using a NanoDrop spectrophotometer (Thermo Scientific). Complimentary DNA (cDNA) was generated using a High Capacity cDNA Reverse Transcription Kit with RNase inhibitors (Multiscribe Reverse Transcriptase. Applied Biosystems) following the manufacturer's protocol. The mRNA level of genes of interest in the sample was determined in triplicate following a real‐time PCR reaction on a 7500 Fast Real‐Time PCR machine for a 10 μL reaction using TaqMan^®^ gene expression assays (20X primers and probe) and Gene Expression Master Mix (2X TaqMan^®^ Universal mix) (all Applied Biosystems). Gene expression was normalized for control gene expression (GAPDH) and calculated according to the ΔΔCT method (Livak & Schmittgen, 2001).

### Immunohistochemistry

Fixed aortic tissue was embedded in paraffin and sectioned at 5 μm thickness (Leica 2255 microtome). A polyclonal antibody to PMCA1 (Sigma‐Aldrich. 1:50 in 5% horse serum, 16 h, 4°C) was detected with an anti‐rabbit horseradish peroxidise‐conjugated secondary antibody (ThermoFisher. 1:200), and colour visualization was obtained by incubating sections in prepared DAB reagent (Dako) for 15 min, with subsequent staining in haematoxylin solution for 20 seconds to stain nuclei.

### Pressure myography

Mesenteric arteries were cleared of adherent tissue and fat and 3rd order arteries isolated and mounted on a pressure myograph (Living Systems Instrumentation, USA), pressurized to an intravascular pressure of 60 mmHg and superfused with physiological salt solution (PSS) of composition (in mm) 119 NaCl, 4.7 KCl, 2.4 MgSO_4_, 25 NaHCO_3_, 1.18 KH_2_PO_4_, 0.07 K_2_EDTA, 6.05 glucose, 1.6 CaCl_2_, aerated with 5% CO_2_/95% air mix and heated to 37°C. Intraluminal diameter (L) and wall thicknesses (W) were continuously measured by video dimension analyser (Hausman *et al*., 2012). Arterial contraction in response to 100 mm potassium solution (high K^+^: K^+^ osmotically replacing Na^+^ in physiological buffer) and subsequent dilation in physiological buffer were recorded. Arteries were superfused with increasing concentration of noradrenaline (NA) and a dose–response curve to the stimulus constructed. Arterial passive properties were measured following superfusion with nominally calcium‐free buffer for 30 min at intravascular pressures of 5–140 mmHg (5, 10, 20 mmHg and subsequently increasing in 20 mmHg steps). Wall thickness to lumen diameter ratio (W:L), cross‐sectional area (CSA), stress, strain and incremental elastic modulus (β) were derived from the recorded diameter and wall thicknesses, as described previously (Hausman *et al*., 2012).

For simultaneous measurement of intracellular free calcium ([Ca^2+^]_i_) and arterial contractility, isolated segments of mesenteric arteries were incubated with 20 μm indo‐1‐AM (Cell Signalling) in HEPES‐buffered physiological solution for 90 min at room temperature and then 30 min at 37°C before being mounted and superfused as described above. The pressure myograph bath was placed atop an inverted microscope, excited at 340 nm and emissions measured via photomultipliers at 400 nm and 500 nm as previously described. Following correction for auto fluorescence the 400:500 nm emission ratio (F_400_/F_500_) was determined. Due to problems associated with calibrating indo‐1 in intact tissues, this ratio was used as an indication of [Ca^2+^]i. Previous work has shown that there is a good agreement between changes in the indo‐1 400:500 nm emission ratio and changes in [Ca^2+^]i in vascular smooth muscle (Austin & Wray, 1995). Basal F_400_/F_500_ levels were compared between groups.

To specifically assess arterial tone, vessels were mounted as described above and superfused in heated PSS for 45 min. Intraluminal pressure was then reduced to 5 mmHg and vessels allowed to equilibrate for 5–10 min as arterial diameter stabilized. Pressure was subsequently increased incrementally to 10, 20, 40, 60, 80 and 100 mmHg (Ca^2+^ containing conditions). Intraluminal pressure was then set to 60 mmHg and arteries were superfused with nominally Ca^2+^‐free buffer for 30 min before pressure was reduced to 5 mmHg and subsequently sequentially increased as previously described (nominally Ca^2+^ free conditions).

### Statistical analysis

Contractile responses are expressed as a percentage contraction to the stimulus relative to the resting lumen diameter in buffer solution. Contraction to NA is plotted as a cumulative dose–response to log[NA]. The log[NA] value for 50% contraction (logEC_50_) was calculated for each artery, and for each genotype group, the mean ± SEM is reported. Intrinsic myogenic tone at each pressure step was derived from the difference in lumen diameter (D) between passive (nominally Ca^2+^ free) and active (Ca^2+^ containing solution) conditions and expressed relative to the passive condition ((D_passive_ – D_active_)/D_passive_). This ratio is presented as a % value of the respective passive condition at the defined internal pressure. The differences between means were considered significant at *P *< 0.05 (*). Data were analysed using GraphPad Prism 5 software. *t*‐test or two‐way ANOVA, with Bonferroni post hoc test, were applied as appropriate. Data were plotted as mean ± SEM; *n *= number of animals.

## Funding

This work was supported by a programme grant from the Medical Research Council UK (G1002082). S. K. Hammad received financial support from the University of Zagazig and A. Njegic from the British Heart Foundation as a PhD candidates during this project.

## Conflict of interest

The authors declare no conflict of interest.

## Author contributions

R. Little, M. Zi, S. Hammad, L. Nguyen, A. Njegic, S. Kurusamy, A. Armesilla, S. Prehar performed the research; R. Little, M. Zi, S. Hammad, A. Njegic analysed the data; C Austin, EJ Cartwright and L Neyses designed the research study; L Neyses, EJ Cartwright and C Austin contributed to acquiring funding; R Little, C Austin and EJ Cartwright contributed to writing the manuscript.

## Supporting information


**Data S1** Supplementary Material.
**Fig. S1** Aged PMCA1^Ht^ mice with elevated blood pressure do not display an adverse cardiac phenotype.
**Fig. S2** Pressure‐lumen diameter relationships significantly differ between active and passive conditions for arteries from WT and PMCA1^Ht^ mice.
