# Supplementary material for: Reduced expression of PMCA1 is associated with increased blood pressure with age which is preceded by remodelling of resistance arteries
Source: Aging Cell. 2017 Aug 9;16(5):1104–13. doi: 10.1111/acel.12637 (PMC5595685; doi:10.1111/acel.12637)
Supplement: Supplementary file 1 — Data S1 Supplementary Material. Fig. S1 Aged PMCA1Ht mice with elevated blood pressure do not display an adverse cardiac phenotype. Fig. S2 Pressure‐lumen diameter relationships significantly differ between active and passive conditions for arteries from WT and PMCA1Ht mice. [file ACEL-16-1104-s001.docx]

SUPPLEMENTARY MATERIAL

**Genotyping**

Ear tissue was digested in a lysis buffer of composition 50mM Tris, 100mM ethylenediaminetetraacetic acid (EDTA) and 0.5% sodium dodecyl sulphate (SDS) with 50µg/mL proteinase K overnight at 56°C. Isolated DNA was precipitated with isopropanol and resuspended in TE buffer (10mM Tris pH 7.5, 1mM EDTA pH 8). Regions surrounding the LoxP sites were amplified using primers (Sigma Genosys) of the following sequences,

Forward: 5’-AGGTGGCTTTAAGACCAGAAACAGGACAGG-3’

Reverse: 5’-CTGTGGAGTACATGCTTCGTTCTGC-3’

at a final concentration of 0.3pM using ReddyMix Extensor PCR master mix (Thermo Scientific AB-0794) in the presence of 0.83mM MgAc. The PCR conditions were 7 minutes 94°C, followed by 30 cycles of 30 seconds 94°C, 30 seconds 65°C, 5 minutes 68°C with final elongation for 10 minutes at 72°C before being held at 4°C. The resulting PCR products were separated on a 0.6% agarose gel containing ethidium bromide and visualised by transillumination (Bio Rad Chemi Doc). The wild type (WT) amplicon was expected at 6662 base pairs (bp) with the homozygous amplicon expected at 4703 bp. No homozygous amplicon was detected alone as the homozygous knock-out is embryonic lethal ([Okunade *et al.* 2004](#_ENREF_37)). Obtaining two bands, for both the WT and knock-out amplicon, indicated a PMCA1 heterozygous genotype.

ADDITIONAL FIGURES

Figure S1:

*Figure S1: Aged PMCA1^Ht^ mice with elevated blood pressure do not display an adverse cardiac phenotype*. **A**. Fractional shortening, **B**. stroke volume, and left ventricular (LV) posterior wall thickness in **C**. systole and **D**. diastole of hearts from 18 month old WT and PMCA1^Ht^ mice is not significantly different (n=8 and 9. T-Test). **E**. Heart weight relative to tibia length (HW/TL) is similar for PMCA1^Ht^ mice compared WT at 18 months old (P=0.718, T-test. n=8 & 9). **F**. Cardiomyocyte cell area is similar for PMCA1^Ht^ mice compared WT at 18 months old (P=0.527, n=4 & 4. T-test). Representative images of haematoxylin and eosin (H & E) stained heart sections used in this calculation shown; scale bar represents 50µm. All data plotted as mean value ± SEM.

Figure S2:

*Figure S2: Pressure-lumen diameter relationships significantly differ between active and passive conditions for arteries from WT and PMCA1^Ht^ mice.* **A & B**. The pressure-diameter relationship in active and passive conditions is significantly different for mesenteric arteries from 6 month old WT (A. n=5) and Ht (B. n=5) mice. **C & D**. The pressure-diameter relationship in active and passive conditions is significantly different for mesenteric arteries from 18 month old WT (C. n=6) and Ht (D. n=6) mice. Non linear regression extra sum of squares F-Test analysis performed. All data plotted as mean value ± SEM. *P<0.05, **P<0.01.
